# Neutrophil-to-Lymphocyte Ratio and Early Neurological Deterioration in Stroke Patients: A Systematic Review and Meta-Analysis

**DOI:** 10.1155/2022/8656864

**Published:** 2022-08-17

**Authors:** Shirin Sarejloo, Hadis Kheradjoo, Sarvin Es Haghi, Samaneh Hosseini, Morad Kohandel Gargari, Amir Azarhomayoun, Shokoufeh Khanzadeh, Shahram Sadeghvand

**Affiliations:** ^1^Cardiovascular Research Center, Shiraz University of Medical Sciences, Shiraz, Iran; ^2^Department of Pathology, Imam Khomeini Hospital, Urmia University of Medical Sciences, Urmia, Iran; ^3^Faculty of Medicine, Shahid Beheshti University of Medical Sciences, Tehran, Iran; ^4^Neurosciences Research Center, Tabriz University of Medical Sciences, Tabriz, Iran; ^5^Tabriz University of Medical Sciences, Tabriz, Iran; ^6^Sina Trauma and Surgery Research Center, Tehran University of Medical Sciences, Tehran, Iran; ^7^Student Research Committee, Tabriz University of Medical Sciences, Tabriz, Iran; ^8^Department of Pediatrics, Tabriz University of Medical Sciences, Tabriz, Iran

## Abstract

In light of the growing emphasis on classifying stroke patients for different levels of monitoring intensity and emergency treatments, we conducted a systematic review of a wide range of clinical studies, according to the preferred reporting items for systematic review and meta-analysis (PRISMA) guidelines, with no restrictions on the language or publication date, to analyze the potential of the neutrophil-to-lymphocyte ratio (NLR) as an early neurological deterioration (END) risk predictor. A comprehensive search was carried out in PubMed, Scopus, and Web of Science databases from the inception to March 13, 2022. Nine articles were included in our study. Stroke patients with END had significantly higher NLR levels than the those without END (SMD = 0.73; CI 95% = 0.42-1.05, *P* value < 0.001). In the subgroup analysis, according to ethnicity, East Asian patients with END had elevated levels of NLR compared to those without END (SMD = 0.79; CI 95% = 0.52-1.06, *P* value < 0.001). However, the difference in the Caucasian group was not significant (SMD = 0.60; CI 95% = −0.50-1.70, *P* value = 0.28). In the subgroup analysis according to the type of stroke, the NLR levels in patients with hemorrhagic stroke who developed END were similar to those without END (SMD = 0.84, CI 95% = −0.10-1.77, *P* value = 0.07). Vice versa, in the ischemic stroke group, patients with END had elevated levels of NLR compared to those without END (SMD = 0.67, CI 95% = 0.38-0.96, *P* value < 0.001). NLR is a unique inflammatory biomarker whose increase in END suggests an immune system dysfunction in the pathogenesis of the disease.

## 1. Introduction

Early neurological deterioration (END) refers to the worsening of symptoms days and weeks after a stroke [1]. Differences in case-mix and END definitions have resulted in a broad range of incidence estimates, with a prior review estimating frequencies ranging from 2.2 to 37.5% at 24 hours after onset [2]. A standard criterion is a two-point increase in the National Institutes of Health Stroke Score (NIHSS) within 24–48 hours after admission [2]. By any definition, the issue of predicting END risk at the time of admission has received considerable critical attention because END is associated with poorer outcomes since it reflects local hemodynamic disruptions, the extension of thrombosis, inflammation, excitotoxicity, and accelerated malfunction of penumbral tissue that covers a compressive hematoma or ischemic core [3]. Radiological and clinical associations with END have already been adequately reported [4]. END has been linked to inflammatory biomarkers such as high-sensitivity C-reactive protein (CRP), leucocyte count, homocysteine, CSF glutamate, and plasma glutamate, according to a recent meta-analysis on stroke biomarkers [5]. However, the neutrophil-to-lymphocyte ratio (NLR) was not taken into account in that meta-analysis.

The NLR is a biomarker that represents the balance between these two elements of the inflammatory response: adaptive immunity (as indicated by the lymphocyte count) and innate immunity (as indicated by the neutrophil count) [6]. It is determined as a simple ratio between the neutrophil and lymphocyte counts assessed in blood samples. NLR has been studied extensively and found to be linked to patient outcomes and disease progression in a range of medical illnesses, including infectious diseases [7], sepsis [8], major cardiac events [9], ischemic stroke [10], and cerebral hemorrhage [10]. Furthermore, greater NLR has been linked to a poor prognosis in cancer patients [11]. These negative connections could be due to the role of severe inflammation and a compromised immune system in the development of these disorders. In the context of stroke, it has been shown that NLR could predict poststroke infection [12], poststroke depression [13], mortality, and functional outcome [14]. By extension, NLR may be a predictor of END in stroke patients. Several studies have found that an elevated NLR is related to the development of END [15–23]. In light of the growing emphasis on classifying stroke patients for different levels of monitoring intensity and emergency treatments, we conducted a systematic review of a wide range of clinical studies, with no restrictions on the language or publication date, to analyze the potential of NLR as an END risk predictor. Although extensive research has been carried out on the relationship between NLR and poor outcome in stroke patients, no single study exists which review the published articles on the potential of NLR as an END risk predictor. This is, to our knowledge, the first meta-analysis on this context. A better knowledge of the link between NLR and END will assist in elucidating the role of inflammation and immunology in the progression and prognosis of this condition, as well as identify patients who require early intervention and further monitoring and imaging.

## 2. Methods

### 2.1. Search Strategy

These systematic review and meta-analysis were performed according to the preferred reporting items for systematic review and meta-analysis (PRISMA) guidelines. A comprehensive search was carried out in PubMed, Scopus, and Web of Science databases from the inception to March 13, 2022. The following keywords were used: (Early neurological deterioration) AND (stroke) AND (neutrophil to lymphocyte ratio OR NLR). References listed in the original reports were searched manually to find any missing articles and further potentially relevant articles.

### 2.2. Study Selection

The inclusion criteria were as follows: (1) case-control or cross-sectional studies, (2) a population including adult patients with stroke, including ischemic and hemorrhagic ones, (3) compared stroke patients with END with those without END, (4) reported the quantitative blood NLR level as mean ± standard deviation (SD) or median (range or interquartile range), and (5) full text being available. The exclusion criteria were as follows: (1) animal studies, review, case reports, comments, or letters; (2) studies that enrolled subjects with any concomitant disorders such as cancer; and (3) duplicate publication.

### 2.3. Data Extraction

The titles and abstracts of the identified studies were evaluated by two authors independently. Potentially, relevant articles identified in the initial assessment were further screened in full text. Data concerning the first author's name, country, year of publication, stroke type, sample size, and NLR levels were extracted independently by two authors. Any discrepancies were resolved by a third reviewer or by discussion.

### 2.4. Quality Assessment

The Newcastle-Ottawa scale (NOS) method with scores ranging from 0 to 9 was adopted to evaluate the quality of the studies [24]. The NOS has three sections: (1) selection of study populations, (2) comparability of populations, and (3) ascertainment of exposure. In the case of any disagreements, the authors would reach a consensus through discussion.

### 2.5. Statistical Analysis

The statistical analysis was conducted using Stata 11.2 software (Stata Corp, College Station, TX). The standard mean difference (SMD) and corresponding 95% confidence interval (CI) were reported. The inconsistency index (*I*^2^) test and Q-statistic were used to assess the heterogeneity across studies. *I*^2^ values of 25%, 50%, and 75% represented small, moderate, and high levels of heterogeneity, respectively. A random effects model would be adopted if significant heterogeneity was found. The method introduced by Wan et al. was adopted to estimate the mean and SD values using the median, range, or IQR values [25]. Subgroup meta-analyses were conducted according to ethnicity (Caucasian and East Asian) and stroke type (ischemic and hemorrhagic). Publication bias was assessed using the funnel plot and Egger test.

## 3. Results

### 3.1. Eligible Studies

**Figure 1:**
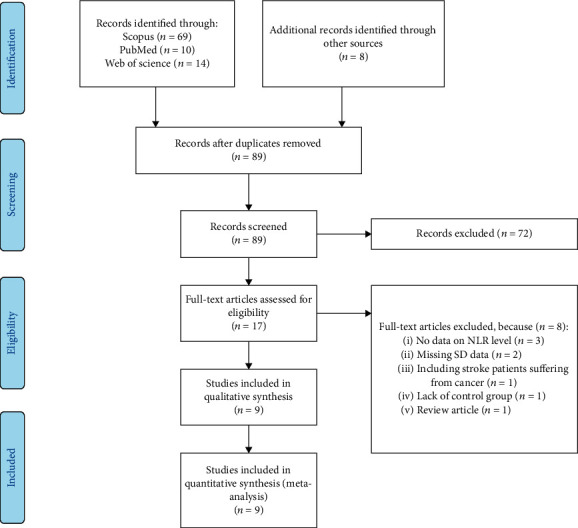
Flow chart of search and study selection.

Of the 101 related studies identified in the initial search, 12 duplicate publications were excluded and 89 remaining articles were screened in title and abstract. Then, 72 studies unrelated to NLR or END were excluded. After screening of the remaining 17 studies in full text, three studies without any data on NLR and END, two studies with missing SD data, one study including stroke patients suffering from cancer, one study without a control group, and one review article were excluded. Finally, nine articles were included in our study [15–23] (Figure 1).

### 3.2. Study Characteristics

All those publications were single-center studies that included adult patients with stroke. The quality score of the study ranged from 5 to 8 according to the NOS. Eight studies were in English and one in Chinese. Three studies were on ischemic stroke and six studies on hemorrhagic stroke. Three studies investigated Caucasian patients, and six studies investigated East Asian patients. One study was prospective, and eight studies were retrospective.  Table 1 shows the characteristics of the included studies.

### 3.3. Overall Meta-Analysis

**Figure 2:**
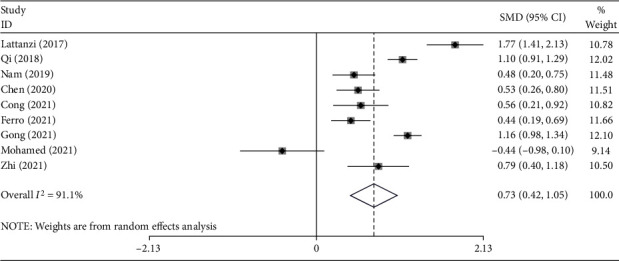
Meta-analysis of differences in NLR level between END and non-END patients.

The nine studies [15–23] reported data belonging to 2724 stroke patients, 742 of whom developed END. The analysis was conducted using the random effects model because of the statistically significant heterogeneity (*I*^2^ = 91.1%, *P* value < 0.0001) (Figure 2). The END group had significantly higher NLR levels than the non-END group (SMD = 0.73; CI 95% = 0.42-1.05, *P* value < 0.001) (Figure 2).

**Figure 3:**
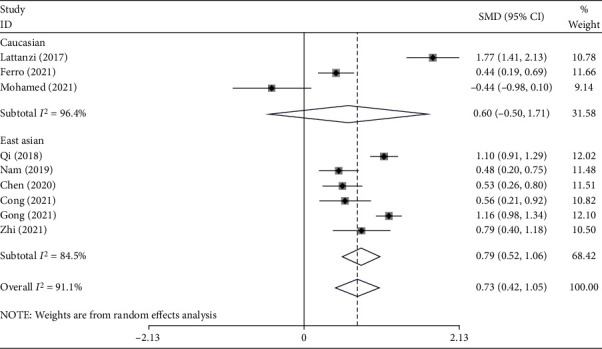
Subgroup analysis of differences in NLR level between END and non-END patients according to ethnicity.

In the subgroup analysis, according to ethnicity, three studies included Caucasian subjects [17, 19, 20], with 597 stroke patients, 156 of whom developed END, and six studies included East Asian subjects [15, 16, 18, 21–23] with 2127 stroke patients, 586 of whom developed END. East Asian patients with END had elevated levels of NLR compared to those without END (SMD = 0.79; CI 95% = 0.52-1.06, *P* value < 0.001). However, the difference in the Caucasian group was not significant (SMD = 0.60; CI 95% = −0.50-1.70, *P* value = 0.28) (Figure 3).

**Figure 4:**
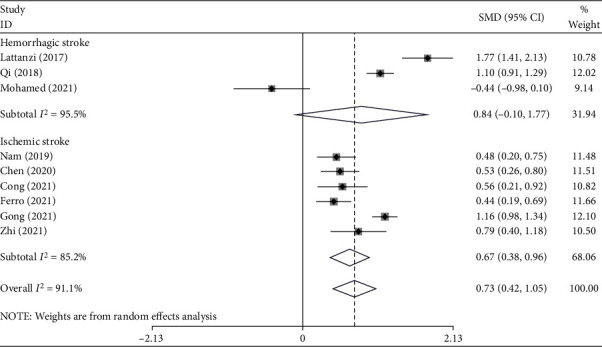
Subgroup analysis of differences in NLR level between END and non-END patients according to type of stroke.

In the subgroup analysis according to the type of stroke, there were three studies on hemorrhagic stroke [19, 20, 22], including 830 patients, of whom 237 developed END, and six studies on ischemic stroke [15–18, 21, 23], including 1894 patients, of whom 505 developed END. The NLR levels in patients with hemorrhagic stroke who developed END were similar to those without END (SMD = 0.84, CI 95% = −0.10-1.77, *P* value = 0.07). Vice versa, in the ischemic stroke group, patients with END had elevated levels of NLR compared to those without END (SMD = 0.67, CI 95% = 0.38-0.96, *P* value < 0.001) (Figure 4).

### 3.4. Publication Bias

**Figure 5:**
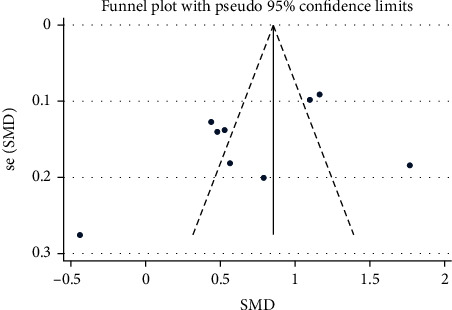
Funnel plot assessing publication bias.

The results of studies on the role of NLR in END showed no publication bias (Egger's test *P* value = 0.13) (Figure 5).

## 4. Discussion

Inflammation is a hallmark of stroke progression and etiology [26]. Inflammation after a stroke is thought to be an essential pathogenic process. The blood-brain barrier (BBB), which was disrupted after the acute brain injury, is passed by stimulated peripheral immune cells such as neutrophils and monocytes, resulting in a variety of negative consequences. Furthermore, the poststroke immune reaction is linked to the severity of the stroke at the time of admission as assessed by the NIHSS. However, the link between poststroke inflammation and END remains unclear. As a result, we conducted this systematic review and meta-analysis to investigate further the impact of inflammatory mediators on END following stroke [26, 27].

The findings of our study show that there was a significant difference in NLR values between stroke patients who developed END and those who did not. NLR values were found to be higher in those who had END. Our findings imply that NLR is a distinct inflammatory marker that may reveal crucial physiologic abnormalities in the development of END.

Severe diseases such as stroke stimulate the generation of neutrophils in the bone marrow and can lead to lymphopenia in a variety of ways [28]. So, relative lymphopenia and neutrophilia can develop, resulting in an increased NLR.

Neutrophils are essential components of innate immunity that help to increase proinflammatory responses [28]. On the other hand, lymphocytes are adaptive immune system components that modulate immunological responses [29]. In the presence of a high NLR, the proinflammatory activity of neutrophils may exceed lymphocytes' regulatory function, creating an environment for uncontrolled peripheral inflammation to spread to a vulnerable brain [30].

In the case of END, a neuroinflammatory pathogenesis hypothesis has gotten a lot of attention [5, 31]. To date, there is strong evidence that neuroinflammation occurs as a result of systemic inflammation. It is postulated that a “crosstalk” occurs between the peripheral inflammatory response and the once “immune-privileged” central nervous system through transportation across the blood-brain barrier (BBB), afferent nerve stimulation, and transmission across circumventricular organs [32]. Uncontrolled peripheral inflammation, assessed by NLR, can take advantage of these pathways to trigger neuroinflammatory processes that lead to END-causing changes in brain systems. For example, new evidence suggests that neutrophils play a significant role in the production of cytokine and chemokine [33], which are commonly raised in END patients and are hypothesized to contribute to neuroinflammation, an essential mechanism in END development and progression [5]. Despite the fact that neutrophils release fewer inflammatory mediators in vitro than their leukocyte counterparts, the neutrophil's cumulative magnitude of secretion, due to their sheer number in circulation, is expected to contribute significantly to cytokine and chemokine pools [33].

CRP, TNF-alpha, IL-4, IL-6, IL-8, and IL-10 are among inflammatory mediators that have been linked to END [5, 34]. IL-6 is currently one of the most researched cytokines in END [35]. It has been shown in numerous research that it is elevated in patients with END [35]. Neutrophils are the main source of IL-6 production in vitro and a primary source of IL-6 in stroke models in rats [33]. Because cytokine secretion may be the result of an unrestrained neutrophilic reaction, normalizing NLR or targeting neutrophils may be an effective therapeutic target.

NLR is a simple biomarker that may be derived during admission from a white blood cell differential and requires no additional time and work. According to recent research, NLR has high sensitivity and specificity for predicting END [15–23]. However, it is worth noting that these figures differ between research, possibly due to differences in the study population and medical circumstances. As a result, in some cases, NLR may be best used in conjunction with other diagnostic markers. Notably, several researchers have constructed prediction models that dramatically boost areas under the receiver operating curves when NLR values are included.

## 5. Limitations

There are a few limitations in our research that must be addressed. To begin with, the study sample size for determining the NLR value in some subgroups, such as Caucasians and patients with hemorrhagic stroke, was limited, and hence, our findings may not be properly powered to draw conclusions about such values. It is also worth noting that NLR values differ by race, which could explain why geographic analysis for NLR had no significant results among Caucasians. It is probable that some populations do not undergo typical hematopoiesis changes after a stroke, and hence, NLR is not useful in those areas. A meta-analysis also carries the risk of study heterogeneity. More than one approach was used to diagnose END among included studies, and, among those used, there is a possibility of user variability due to their subjective nature. As a result, these studies may have varied rates of missed diagnoses, which could compromise their validity.

## 6. Conclusion

The findings of our investigation support a link between NLR levels and the development of END in stroke patients. NLR is a unique inflammatory biomarker whose increase in END suggests an immune system dysfunction in the pathogenesis of the disease. Furthermore, our data suggest that NLR is a viable biomarker that may be easily introduced into clinical settings to help predict and prevent END. Finally, the development of new therapeutic modalities and biomarkers will help us better prevent and treat END, lowering long-term mortality and morbidity.

## Figures and Tables

**Table 1:** General characteristics of included studies.

First author	Year	Country	Type of stroke	Ethnicity	Design	END definition	END group		Non-END group		NOS score
							*N*	NLR	*N*	NLR	
Lattanzi et al. [19]	2017	Italy	Hemorrhagic	Caucasian	R	(1) An increase ≥ 4 on the NIHSS score, (2) a decrease ≥ 2 on GCS, or (3) death occurred from the time of admission to 7 days posthemorrhage	54	9.46 ± 5.80	138	3.28 ± 1.98	8
Qi et al. [22]	2018	China	Hemorrhagic	East Asian	R	(1) An increase ≥ 4 on the NIHSS score, (2) a decrease ≥ 2 on GCS, or (3) death occurred from the time of admission to 7 days posthemorrhage	166	15.98 ± 8.83	392	8.03 ± 6.44	7
Nam et al. [21]	2019	Korea	Ischemic	East Asian	R	An increase ≥ 2 on the total NIHSS score or ≥1 on the motor NIHSS score within the first 72 hours of admission	63	3.49 ± 2.73	286	2.56 ± 1.72	7
Chen et al. [15]	2020	China	Ischemic	East Asian	R	(1) An increase in NIHSS ≥ 4 from baseline or (2) an increase in Ia of NIHSS ≥ 1 within 72 h	77	8.06 ± 8.36	180	5.24 ± 3.28	7
Cong and Ma [16]	2021	China	Ischemic	East Asian	R	(1) An increase > 2 points in the total NIHSS score compared to the score at admission, (2) an increase > 1 point in the NIHSS specificity subitems, namely, level of consciousness (1a–1c) or motor capacity (5a–6b), or (3) new neurological deficits despite no change in the NIHSS score	55	6.06 ± 8.36	74	4.16 ± 3.73	8
Ferro et al. [17]	2021	Portugal	Ischemic	Caucasian	R	An increase in NIHSS at 24 hours from the baseline	85	6.83 ± 5.81	240	6.05 ± 4.95	8
Gong et al. [18]	2021	China	Ischemic	East Asian	R	An increase in the NIHSS score by ≥4 points in the total score within 24 h after thrombolysis	193	8.42 ± 6.50	469	4.18 ± 1.57	7
Mohamed et al. [20]	2021	Egypt	Hemorrhagic	Caucasian	P	(1) An increase ≥ 4 on the NIHSS score, (2) a decrease ≥ 2 on GCS, or (3) death occurred from the time of admission to 7 days posthemorrhage	17	15.92 ± 6.86	63	23.25 ± 18.39	8
Zhi and Xiaopeng [23]	2021	China	Ischemic	East Asian	R	An increase ≥ 2 on the total NIHSS score or ≥1 on the motor NIHSS score within the first 72 hours of admission	32	3.30 ± 1.70	140	2.40 ± 0.97	5

R: retrospective; P: prospective; *N*: number; SD: standard deviation; NLR: neutrophil to lymphocyte ratio; NOS: Newcastle-Ottawa scale.

## Data Availability

All data generated or analyzed during this study are included in this published article.
